# Price, familiarity, and availability determine the choice of drug - a population-based survey five years after generic substitution was introduced in Finland

**DOI:** 10.1186/1472-6904-11-20

**Published:** 2011-12-15

**Authors:** Reeta Heikkilä, Pekka Mäntyselkä, Riitta Ahonen

**Affiliations:** 1 University of Eastern Finland, Faculty of Health Sciences, School of Pharmacy, Social Pharmacy, P.O.Box 1627, FI-70211 Kuopio, Finland; 2 University of Eastern Finland, Faculty of Health Sciences, School of Medicine, Department of Primary Health Care, P.O.Box 1627, FI-70211 Kuopio, Finland; 3 Kuopio University Hospital, Unit of Primary Health Care, P.O.Box 1777, FI-70211 Kuopio, Finland

## Abstract

**Background:**

Mandatory generic substitution (GS) was introduced in Finland at the beginning of April 2003. However, individual patients or physicians may forbid the substitution. GS was a significant change for Finnish medicine users. It was thought it would confuse people when the names, colors, packages, etc., changed. The purpose of this study was to explore what medicine-related factors influence people's choice of prescription drugs five years after generic substitution was introduced in Finland.

**Methods:**

A population survey was carried out during the autumn of 2008. A random sample was drawn from five mainland counties. A questionnaire was mailed to 3000 people at least 18 years old and living in Finland. The questionnaire consisted of both structured and open-ended questions. Factors that influenced the subjects' choice of medicines were asked with a structured question containing 11 propositions. Descriptive statistical analyses were performed.

**Results:**

In total, 1844 questionnaires were returned (response rate, 62%). The percentage of female respondents was 55%. Price, availability, and familiarity were the three most important factors that influenced the choice of medicines. For the people who had refused GS, the familiarity of the medicine was the most important factor. For the subjects who had allowed GS and for those who had both refused and allowed GS, price was the most important factor.

**Conclusions:**

The present study shows that price, familiarity, and availability were important factors in the choice of prescription medicines. The external characteristics of the medicines, for instance the color and shape of the tablet/capsule or the appearance of the package, were not significant characteristics for people.

## Background

Generic substitution (GS) was introduced in Finland at the beginning of April 2003 with the aim of curbing the rise in medical expenses for society and individuals. The reform was preceded by heated public debate. For example, the Finnish Medical Association and the pharmaceutical industry (Pharma Industry Finland) objected to it because, for example, they were afraid of decreasing adherence [1,2]. It was thought that generic substitution would confuse people when the names, colors, packages and other physical appearance of drug products changed. It is certain that generic substitution was a considerable health policy reform for Finnish medicine users. People were not used to making decisions related to their medication. Before generic substitution, a medicine could be changed to another product in the pharmacy only after consultation with the physician. The reform places the dispensing pharmacy under an obligation to substitute a medical product, prescribed by a physician or dentist, with the cheapest, or close to the cheapest, interchangeable product. However, the prescriber or individual patients may forbid the substitution. Individual patients can forbid GS at any time and the prescriber can forbid GS for medical or therapeutical reasons.

Previous studies have found some factors related to patient involvement in nonprescription medicine purchasing [3-6]. For example, higher educational level or higher family income has caused the lower involvement in nonprescription medicine purchase decisions [3]. Also a recommendation by pharmacists [5], effectiveness, familiarity with the name or brand and safety have influenced nonprescription medicines purchasing [4]. In addition, people's previous experiences of nonprescription medicines influence their decision later [6]. However, we did not know about medicine-related factors that influence patients' choice of prescription drugs.

Generic medicines have caused certain problems in many countries [7-14]. According to an Australian study (n = 204), patients (average age 72 years) were confused most frequently (56%) by generic and trade names, while poor adherence was reported by 53% [15]. Also, a new Swedish study reported that 40% of respondents reported at least one difficulty related to generic medicines and substitution. There was inconsistent information about the effects of generic substitution (GS) on adherence in patients who used antihypertensive drugs. According to a Netherlands study based on prescription data and hospital discharge records, generic substitution of hypertensive drugs did not lead to lower adherence compared with brand name drugs [16]. Neither was there any difference in hospitalizations for cardiovascular diseases in the six months after the substitutions were observed. In a Norwegian interview study, generic substitution affected adherence because patients were uncertain about the difference between old and new products [14].

This study was one part of a larger study exploring the risks and benefits of generic substitution in Finland. More details on generic substitution in Finland are described in our previous studies [17,18]. According to our best knowledge, there are no published population surveys dealing with factors related to medicines' influence on people's choice of prescription drugs. In the present study we were especially interested in the differences between people who had refused GS and those who had allowed GS.

The aim of this present study was to explore what factors related to medicines influence people's choice of prescription drugs five years after generic substitution was introduced in Finland.

## Methods

**Figure 1:**
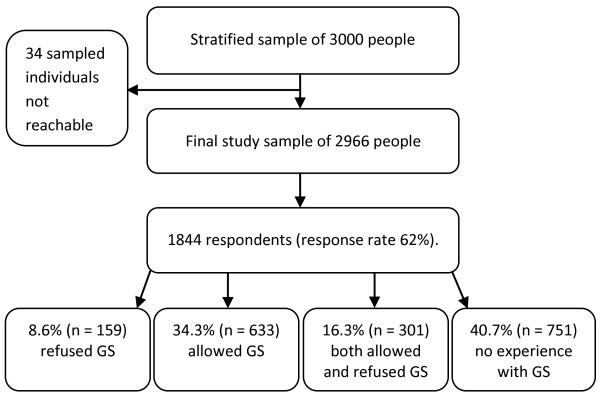
**Flow chart of the postal survey process**.

This population survey was carried out during the autumn of 2008. A random sample was drawn from five mainland counties: Southern Finland, Eastern Finland, Western Finland, Oulu, and Lapland. The sixth county, Åland, was excluded from the sample because of its divergent drug use culture compared with the other counties [19]. We wanted to include in the sample individuals who had substituted their medicines, individuals who had refused substitution, and also those who had no experience with GS. According to the register of The Social Insurance Institution of Finland, in 2008 only 10% of Finnish pharmacy customers refused GS. Because the proportion was so small, we wanted to make sure we included enough individuals who had refused GS in the sample. Therefore, we obtained statistics, by hospital district, about people who had refused GS during 2007 from The Social Insurance Institution of Finland. The hospital districts were located in the counties. In 2007 altogether 778,902 individuals (excluding Åland) refused generic substitution in Finland. Of these, 38% lived in Southern Finland, 11% in Eastern Finland, 40% in Western Finland, 7% in Oulu, and 4.5% in Lapland. The random sample (n = 3000) was formulated on the grounds of these percentage values. So, the sample included 1140 persons from Southern Finland, 340 persons from Eastern Finland, 1190 persons from Western Finland, 220 persons from Oulu, and 110 persons from Lapland. A flow chart of the postal survey process is presented in Figure 1.

A questionnaire was mailed to a random sample of 3000 people at least 18 years old and living in Finland. The sampling was conducted by the Finnish Population Register Centre from their database. Two reminders were sent after the first mailing round. The first page of the questionnaire was meant for all respondents. The second page was meant for respondents who had substituted their medicines at least once and the third page was meant for respondents who had refused substitution at least once. The last page was reserved for free comments. The questionnaire consisted of both structured and open-ended questions. The questionnaire was developed on the basis of our previous study of pharmacy customers in 2003 [17], when GS was introduced in Finland. The questionnaire was piloted the first time in 2003 and again in 2008 after editing. Factors that influenced the subjects' choice of medicines were asked with a following question "Which of the following are important when you are choosing a medication." The question contained 11 propositions that were price, availability, familiarity, domestic product, excipients, shape of the tablet/capsule, color of the tablet/capsule, appearance of the package, manufacturer, splittability, and brand name. The respondents could choose (circle) more than one proposition. The propositions were almost the same as in the earlier study. However, we added three propositions (splittability, manufacturer, brand name) that emerged in the earlier study. Background information (sex, year of birth, county) were asked with structured questions. Use of prescription drugs was asked with the question "Do you use one or more prescription drugs regularly?"

The study setting and the complete anonymity of the respondents were in accordance with the local ethical instructions for researchers. In Finland, questionnaire studies are not required to be approved by the ethics committee. The ethics committee states that respondents give their approval when they answer the questionnaire.

The data were analyzed with SPSS 17.0.1 statistical software (SPSS Inc. Chicago, IL) using frequencies and cross-tabulations for descriptive analysis.

## Results

Thirty-four questionnaires did not reach the recipients for various reasons (e.g. addresses were wrong; the intended recipient had moved abroad; death) or because the recipients were excluded due to poor health or institutional care. A total of 1844 of the remaining 2966 questionnaires were returned (response rate, 62%). The percentage of female respondents was 55%. The mean age of the respondents was 54 years and their median age was 55 years. The detailed characteristics of the study population are presented in Table 1.

**Table 1:** Summary of study population

	Total		Patients who refused GS		Patients who allowed GS		Patients who had refused and allowed GS		Patients who had no experience with GS	
	**No**.	**(%)**	**No**.	**(%)**	**No**.	**(%)**	**No**.	**(%)**	**No**.	**(%)**

**All**	1844	(100)	159	(9)	633	(34)	301	(16)	751	(41)
**Sex**										
**Male**	826	(45)	54	(34)	254	(40)	130	(43)	388	(52)
**Female**	1018	(55)	105	(66)	379	(60)	171	(57)	363	(48)
**Age, yr**										
**18-59**	1118	(61)	70	(45)	404	(64)	154	(52)	490	(66)
**60-94**	706	(39)	87	(55)	227	(36)	145	(48)	247	(34)
**Not reported**	20		2		2		2		14	
**Mean (SD)**	54	(17)	61	(18)	53	(16)	57	(19)	52	(17)
**Median**	55		62		53		59		52	
**Regularly uses prescription drugs**										
**Yes**	1085	(59)	140	(88)	440	(70)	239	(80)	266	(36)
**No**	748	(41)	19	(12)	190	(30)	60	(20)	479	(64)
**Not reported**	11		0		3		2		6	

Price (72%), familiarity (56%), and availability (42%) were the three most important factors that influenced the choice of medicines (Table 2). Other characteristics of the medicines, such as domestic product (25%), splittability (24%), excipients (16%), manufacturer (10%), brand name (8%), shape of the tablet/capsule (6%), color of the tablet/capsule (1%), and appearance of the package (1%), were not as important factors to the respondents.

**Table 2:** Three most important factors that influence the choice of prescription medicines

			Factors related to medicines					
			Price		Familiarity		Availability	
		**No**.	**No**.	**%**	**No**.	**%**	**No**.	**%**

**All respondents**		1551	1112	(72)	874	(56)	643	(42)
Gender	Men	688	492	(72)	366	(53)	298	(43)
	Women	863	620	(72)	508	(59)	345	(40)
Prescription medicines in	Yes	919	633	(69)	474	(52)	403	(44)
regular use	No	624	472	(76)	396	(64)	237	(38)
Age, years	< 60	952	742	(78)	554	(58)	402	(42)
	≥ 60	585	363	(62)	314	(54)	230	(39)
**Patients who had refused GS**								
All		134	60	(45)	95	(71)	50	(37)
Gender	Men	45	21	(47)	32	(71)	15	(33)
	Women	89	39	(44)	63	(71)	35	(39)
Prescription medicines in	Yes	117	52	(44)	85	(73)	44	(38)
regular use	No	17	8	(47)	10	(59)	6	(35)
Age, years	< 60	63	36	(57)	45	(71)	23	(37)
	≥ 60	70	24	(34)	49	(70)	26	(37)
**Patients who had allowed GS**								
All		538	448	(83)	242	(45)	203	(38)
Gender	Men	212	170	(80)	87	(41)	84	(40)
	Women	326	278	(85)	155	(48)	119	(37)
Prescription medicines in	Yes	380	308	(81)	159	(42)	151	(40)
regular use	No	155	137	(88)	82	(53)	50	(32)
Age, years	< 60	340	297	(87)	159	(47)	133	(39)
	≥ 60	196	150	(77)	83	(42)	69	(35)
**Patients who had refused and allowed GS**								
All		251	178	(71)	137	(55)	119	(47)
Gender	Men	108	81	(75)	46	(43)	52	(48)
	Women	143	97	(68)	91	(64)	67	(47)
Prescription medicines in	Yes	200	137	(69)	103	(52)	103	(52)
regular use	No	49	39	(80)	32	(65)	16	(33)
Age, years	< 60	124	101	(82)	76	(61)	58	(47)
	≥ 60	125	76	(61)	60	(48)	60	(48)
**No experience**								
All		628	426	(68)	400	(64)	271	(43)
Gender	Men	323	220	(68)	201	(62)	147	(46)
	Women	305	206	(68)	199	(65)	124	(41)
Prescription medicines in	Yes	222	136	(61)	127	(57)	105	(47)
regular use	No	403	288	(72)	272	(68)	165	(41)
Age, years	< 60	425	308	(73)	274	(65)	188	(44)
	≥ 60	194	113	(58)	122	(63)	75	(39)

Respondents could choose several options.

For the people who had refused GS, the familiarity of the medicine was the most important factor that influenced their choice of medicine (Table 2). People who used prescription medicines regularly valued that characteristic more often than patients who did not use prescription medicines regularly.

People who had allowed GS considered price the most important characteristic in their choice of medicine, in contrast to people who had refused GS (Table 2). Price was a very important characteristic especially for women, for people who did not use any prescription medicine regularly, and for people under 60 years of age.

People who had both refused and allowed GS also appreciated price as the most important characteristic (Table 2). People who did not use any prescription medicines regularly considered price more important than people who used prescription medicines regularly. Also, people under 60 years of age considered price a very important characteristic.

For the respondents who had no experience with GS, price and familiarity were the most important characteristics in their choice of medicines.

## Discussion

According to our study, price, familiarity, and availability were the three most important factors that influenced the choice of prescription medicines. People who allowed GS or who had both refused and allowed GS considered price the most important factor. People who had refused GS considered familiarity much more important than price. People who had no experience at all with GS appreciated both of these factors. The fact that price was the most important factor for people is in line with our earlier study, where savings were the main reason for accepting GS [17,18]. Finnish people also have confidence in the effect of cheaper medicines [18]. They also held the opinion that GS does not cause any risk to drug safety.

The people who had refused GS were a little older than the people in the other groups. It is obvious that especially older people appreciated the familiarity and price of medicines. Many old people often have someone else buy their medicines for them. If the possibility of GS had not been discussed before, it is probable that the representative would refuse GS. However, if the representative allows GS, it is possible that he/she cannot take into account the familiarity of the medicine to the old person being represented. Before GS was introduced it was thought it would confuse patients when the names, colors, packages, etc., of drug products changed [1,2]. Although people did not usually choose prescription medicines on the grounds of these external characteristics of medicines, confusions could be possible. The Finnish Medicines Agency, Fimea, publishes an interchangeable drug list quarterly. For example, in 2008 (list July 2008-September 2008) the list included 23 simvastatin products (20 mg), 39 mirtazapine products (15 mg), and 23 amlodipine products (10 mg). While pharmacies cannot stock all these interchangeable products, there still are many drug products to choose from.

The response rate of 62% was quite good, since the present study was based on a population survey and the sample also included individuals who did not know what GS is. The response rate was 67% for women and 56% for men. The difference between sexes, in proportion and response rate, was similar in a previous Finnish population survey [20-22]. The age distribution of the respondents was quite similar to the age distribution of the sample and background population. Young age groups were somewhat under-represented among the respondents. Young people usually use less medicine than older age groups, and maybe for that reason they were not motivated to answer our questionnaire. Also, the proportion of men (45%) and women (55%) respondents was quite well in line with the sample (men 49%, women 51%) and the population (men 49%, women 51%).

## Conclusions

Price, familiarity, and availability were important factors in the choice of prescription medicines. The external characteristics of medicines, for instance the color and shape of the tablet/capsule or the appearance of the package, were not significant characteristics for people.

## Competing interests

The authors declare that they have no competing interests.

## Authors' contributions

RH designed and carried out the postal survey and drafted the manuscript. PM participated in the design of the postal survey and helped draft the manuscript. RA participated in the design of the postal survey and helped draft the manuscript. All the authors read and approved the final manuscript.

## Pre-publication history

The pre-publication history for this paper can be accessed here:

http://www.biomedcentral.com/1472-6904/11/20/prepub

## References

[B1] Finnish Medical Association Lausunto luonnoksesta hallituksen esitykseksi eduskunnalle lääkelain ja sairausvakuutuslain muuttamiseksi. Statement for the Ministry of Social Affairs and Health 2002 [in Finnish]

[B2] Pharma Industry Finland Lausunto luonnoksesta lakiehdotukseksi geneerisen substituution toteuttamiseksi Suomessa. Statement for the Ministry of Social Affairs and Health 2002 [in Finnish]

[B3] Gore P Madhavan S McClung G Riley D Consumer involvement in nonprescription medicine purchase decisions J Health Care Mark 1994 14 16 23 10137123

[B4] Hanna LA Hughes CM Public's views on making decisions about over-the-counter medication and their attitudes towards evidence of effectiveness: A cross-sectional questionnaire study Patient Educ Couns 2011 83 345 351 10.1016/j.pec.2011.02.016 21440405

[B5] Wazaify M Shields E Hughes CM McElnay JC Societal perspectives on over-the-counter (OTC) medicines Fam Pract 2005 22 170 176 10.1093/fampra/cmh723 15710640

[B6] The Proprietary Association of Great Britain A Summary Profile of the OTC Consumer 2005 London

[B7] Andersen ML Laursen K Schaumann M Rubak SL Olesgaard P Mainz J Lauritzen T How do patients evaluate the newly introduced system of substituting prescriptions? Ugeskr Laeger 2000 162 6066 6069 [in Danish] 11107943

[B8] Rubak SL Andersen ML Mainz J Olesgaard P Lauritzen T How do practitioners evaluate the newly introduced system of substituting prescriptions? Ugeskr Laeger 2000 162 6070 6073 [in Danish] 11107944

[B9] Rubak SL Andersen ML Mainz J Olesgaard P Laursen K Schaumann M Lauritzen T How do pharmacists evaluate the newly introduced system of substituting prescriptions? Ugeskr Laeger 2000 162 6074 6077 [in Danish] 11107945

[B10] Socialstyrelsen Patientsäkerhet vid utbyte av läkemedel på apotek 2004 Stockholm [in Swedish]22224173

[B11] Frisk P Rydberg T Carlsten A Ekedahl A Patients' experiences with generic substitution: a Swedish pharmacy survey Journal of Pharmaceutical Health Services Research 2011 2 9 15 10.1111/j.1759-8893.2011.00036.x

[B12] Gill L Helkkula A Cobelli N White L How do customers and pharmacists experience generic substitution? International journal of pharmaceutical and healthcare marketing 2010 4 375 395

[B13] Håkonsen H Toverud EL Special challenges for drug adherence following generic substitution in Pakistani immigrants living in Norway Eur J Clin Pharmacol 2011 67 193 201 10.1007/s00228-010-0960-9 21161197 PMC3021708

[B14] Toverud EL Roise AK Hogstad G Wabo I Norwegian patients on generic antihypertensive drugs: a qualitative study of their own experiences Eur J Clin Pharmacol 2011 67 33 38 21104408 10.1007/s00228-010-0935-x PMC3016237

[B15] Sorensen L Stokes JA Purdie DM Woodward M Roberts MS Medication management at home: medication risk factor prevalence and inter-relationships J Clin Pharm Ther 2006 31 485 491 10.1111/j.1365-2710.2006.00768.x 16958827

[B16] Van Wijk BL Klungel OH Heerdink ER de Boer A Generic substitution of antihypertensive drugs: does it affect adherence? Ann Pharmacother 2006 40 15 20 16303985 10.1345/aph.1G163

[B17] Heikkilä R Mäntyselkä P Hartikainen-Herranen K Ahonen R Customers' and physicians' opinions of and experiences with generic substitution during the first year in Finland Health Policy 2007 82 366 374 10.1016/j.healthpol.2006.10.006 17141355

[B18] Heikkilä R Mäntyselkä P Ahonen R Do people regard cheaper medicines effective? Population survey on public opinion of generic substitution in Finland Pharmacoepidemiol Drug Saf 2011 20 185 191 10.1002/pds.2084 21254290

[B19] Lahnajärvi L Klaukka T Enlund H Ahvenanmaa - itsehallittua lääkekulutusta 1997 Helsinki: Kansaneläkelaitos

[B20] Ylinen S Hameen-Anttila K Sepponen K Lindblad AK Ahonen R The use of prescription medicines and self-medication among children-a population-based study in Finland Pharmacoepidemiol Drug Saf 2010 19 1000 1008 10.1002/pds.1963 20712023

[B21] Turunen JH Mäntyselkä PT Kumpusalo EA Ahonen RS Frequent analgesic use at population level: prevalence and patterns of use Pain 2005 115 374 381 10.1016/j.pain.2005.03.013 15911164

[B22] Pohjanoksa-Mäntylä M Bell JS Helakorpi S Närhi U Pelkonen A Airaksinen MS Is the Internet replacing health professionals? A population survey on sources of medicines information among people with mental disorders Soc Psychiatry Psychiatr Epidemiol 2011 46 373 379 10.1007/s00127-010-0201-7 20225134

